# Separating the Wheat From the Chaff: Guidance From New Technologies for Detecting Deception in the Courtroom

**DOI:** 10.3389/fpsyt.2018.00774

**Published:** 2019-01-17

**Authors:** Judee K. Burgoon

**Affiliations:** Center for the Management of Information, University of Arizona, Tucson, AZ, United States

**Keywords:** lying, deception detection, linguistics, multimodal deception, nonverbal deception

The courtroom is among the most challenging contexts for detecting deception. Testimony has been carefully scripted and rehearsed in advance. Witnesses may proffer answers that create rather than reduce ambiguity. Questioning may skew attention away from a defendant's transgressions. Character testimony may malign opposition witnesses while painting a sanitized picture of the defendant. Amid so many inconsistent depictions, facts and opinions, jurists face a significant challenge in separating valid wheat from invalid chaff.

Jurists currently must render decisions unaided by the latest lie detection technologies such as fMRI (1), which places respondents in a loud, magnetized tube; EEG, ERP or fNIRS (2, 3), which connect wires to respondents' head or hands to detect brain waves; computer vision techniques that extract facial expressions from videotapes (4); instruments that discern voice pitch, tempo and fluency from audio recordings (5); or software that identifies linguistic patterns (6). Or, questioning techniques like the Concealed Information Test (7) and the autobiographical Implicit Association Test (8), in which respondents are questioned about aspects of a crime while their response latency is gauged. Jurists must rely on their own observational acumen, what they see and hear.

Nevertheless, we can learn what these technologies and techniques have unearthed that is applicable to courtroom deceit, focusing especially on indicators that prevaricators are less likely to attend to or control. Here deceit references the whole gamut of what is said, not said, and how it is said, both non-verbally and verbally. There is no silver bullet, no single indicator, that will invariably gauge a speaker's veracity (9), but by taking a holistic approach that bundles indicators together (10) and combines them across modalities (11) and by looking for temporal changes from baseline to later responding (12), it is possible to improve detection accuracy over that of the unaided human jurist (13).

## Non-verbal Signals

The various non-verbal indicators of deceit can be grouped according to what they signify: (1) arousal and emotional activation, (2) cognitive difficulty, (3) memory access, (4) attempted control of unbidden behavior, and (5) self-presentation (14, 15).

### Emotions and Arousal

The first avenue of spotting telltale signs has been to look for outward signs of anxiety, fear, shame and other negative emotions (16). These are thought to be involuntary and uncontrollable or uncontrolled autonomic responses. Microexpressions of emotions such as contempt have been touted as reliable (17). But, among the many shortcomings of microexpressions is that they are not readily observable at normal courtroom interaction distances and are extremely infrequent [see (18)]. Better to watch for *macro*-expressions (19), which can leak feigned sadness or inappropriately felt happiness, especially during high-intensity fear (20, 21). However, because people work to manage their facial expressions, these are often not the best place to look.

More helpful are some indicators of arousal [(22); but cf. (4)]. Subtle behaviors like restive foot movements and postural shifts reveal unease but may not be visible when suspects and witnesses take the stand. Close up one might be able to observe blinks and pupil dilation, which are tied to arousal (23). But, absent videotaped recordings available for slow-motion review, these would be difficult to spot in the courtroom. More visible are what I have labeled face-adaptors and lip-adaptors—behaviors that function to alleviate discomfort. The former are things like rubbing one's check or neck and twisting hair. The latter are lip movements such as biting, licking, scrunching and tongue-showing that are associated with states of nervousness or serious concentration. These are less likely to be controlled by liars.

Also telling are vocal indicators: higher voice pitch, increased vocal tension, and more hesitations and speech errors (24). The fallacy of relying too heavily on arousal and emotion indicators is that such arousal behaviors are not associated exclusively with deception. Witnesses and innocent suspects may exhibit these indicators just because testifying in a courtroom is anxiety-inducing, resulting in them being judged deceptive—a false-positive—whereas guilty suspects may be sufficiently coached and rehearsed as to be judged truthful—a false-negative. A jurist's level of discernment must be highly tuned to navigate between the revealing and the concealing signals.

### Cognitive Difficulty

Many scholars have argued that a more fruitful direction in identifying valid and reliable indicators is to focus not on misleading signs from affect but on cognitive difficulty (25). These indicators derive from the assumption that lying is harder than truth-telling and will produce outward signs of such difficulty [(26); but cf. (27)]. Kinesic behaviors include blinks, avoidance of eye contact, reduction in illustrator gestures, and cessation of gesturing. Vocalic indicators include delayed responding to questions, shorter responses, and more speech errors (5, 24). All of these indicators are detectable in the courtroom and are among the most reliable ones available. Questioning that would be easy for truth-tellers to answer but difficult for deceivers (e.g., Who else might have had reason to commit X?) are most likely to elicit them.

### Mental Processes

An extension of the cognitive approach is to consider what memory processes are implicated with lying. Liars engaged in serious lies—the type present in courtrooms—not only must access the truth and decide *if* to lie, but also conduct a cost-benefit analysis of different forms of deceit, choose the type of lie to be expressed, decide how to enact the lie non-verbally and verbally, and anticipate receivers' responses (28). A meta-analysis by Christ et al. (29) established that lying entails 8 of 13 brain regions and 173 deception-related foci that are more active for deceptive than truthful responses. These included accessing working memory, inhibitory control, and task switching (i.e., interspersing truthful with deceptive details). These mental gymnastics need not entail extreme mental effort to produce indicators of these executive processes. Longer between-turn and within-turn pauses (30) along with non-fluencies, gaze aversion and temporary cessation of gestures are likely to be most relevant. Again, these indicators may be indistinguishable from other cognitive difficulty indicators, so it becomes essential to evaluate the nature and difficulty of the questions they answer.

### Behavioral Control

The aforementioned research frequently points to liars reducing postural, gestural, head and facial activity to the point of crossing a thin line between appearing composed and appearing wooden, rigid and unnatural (31). This generalized inhibition and rigidity across trunk, limbs, head, and face may reflect overcontrol of felt arousal and negative emotions (32, 33). Even when told about this trend, liars fail to increase their movement (34). Thus, attempted control does not succeed fully and may be one of the best classes of deception indicators because truth-tellers, in an effort to maximize their credibility, are likely to become more, not less, animated.

### Self-Presentation

Scholars and practitioners alike have opined that deceivers attempt to project a demeanor of honesty and believability. This is more likely to occur when deceivers have opportunities to plan, rehearse, or adapt how they appear and sound (35). Especially they may adopt a veneer of facial and vocal pleasantness and calm. In the courtroom, judgments must factor into account the likelihood that witnesses and suspects are well-practiced in responding to anticipated questions. Smooth, fluent presentations therefore may or may not be indicative of truthfulness. The longer respondents are on the stand, the more they will be able to detect jurors' belief in their testimony and adapt responses accordingly. Veracity judgments formed early should be more informative than ones formed later.

## Verbal Signals

Turning next to automatically analyzed linguistic indicators, seven clusters taken from Burgoon et al. (36) are likely to matter in the forensic context.

### Quantity and Specificity

Deceivers tend toward shorter statements (fewer words and phrases) and less specific sensory, spatial and temporal details (37, 38). But when respondents are highly motivated and when accounts have been rehearsed over and over, this difference may evaporate (36). Here is where questioning strategies that elicit specific details can challenge liars while aiding truth tellers with their recall. “Was it daylight or twilight?” “What did the immediate vicinity look like?” And so on. Asking respondents to take a second look from a different perspective—perhaps the viewpoint of a bystander—has two advantages (39). First, deceivers who are fabricating an account will not have new details to present and may fear that inventing new ones risks contradicting previous statements, a risk compounded by any mental strain they are experiencing. Second, truth-tellers are often eager to be helpful, even adopting a Sherlock Holmes or Agatha Christie mantle of offering yet more recalled details.

### Diversity

A key tip-off of veracity is how varied a speaker's vocabulary is. This feature is somewhat beyond liars' control. They can't spontaneously broaden their lexicon. And deceivers are especially likely to repeat the same phraseology over and over. In the face of deeper questioning, liars' primary strategy is to stick to their same prepared cover-story, whereas truth tellers principally try to be honest, leading to more varied responding (40). Repetitiveness is thus less common among truth-tellers.

### Ambiguity/Hedging/Uncertainty

Vagueness, equivocation and hedging language such as weak modal verbs (“might have”), tentative words (“maybe”), and passive voice (“Mistakes were made”) are more common in fraudulent statements (37, 38, 41), especially during unprepared remarks (36). The caveat is that liars may also pepper their remarks with linguistic markers of certainty to project confidence.

### Personalism

This is a tricky one because researchers and practitioners have pointed to the “I” and “me” personal pronouns for indications of whether or not speakers take ownership of what they are saying. Liars recounting an accused rape may use personal pronouns (“I did this,” “I did that”) until the time frame of the actual event then shift to impersonal language. But this indicator varies wildly across written statements, interviews and in-person communication, making it unreliable; second person (“you”) pronouns and impersonal pronouns have an equally checkered record (42).

### Immediacy

Responding in the “here and now” (using linguistic immediacy) is often associated with truthfulness and non-immediacy, with deceit (43). However, that doesn't always hold in the courtroom. Shifts in verb tense from past to present (“I *go* golfing” instead of “I *went* golfing”) produce less precision and more uncertainty in answer to the question, “Where were you last weekend?” In other cases, more immediate language is associated with truthfulness. Parents of missing children who fraudulently appeal for the return of their already-dead children may make statements like, “She *was* such a sweet girl.” The validity of language immediacy as a veracity indicator depends on whether verb tense matches what is expected. For example, the question, “What did you do next?” calls for past tense; the question, “What are you thinking right now?” calls for present tense. If the tense is a mismatch with the question, it warrants a deeper dig. Amount of advance time for planning one's account can also increase the immediacy of language (44).

### Emotion

Here I am talking about whether the language in use carries emotional overtones. This is also an indicator with an irregular history. It has been proposed in some quarters [e.g., (45)] that deceivers' fear, guilt, and shame creep into their choice of language. There is good evidence that compared to truth-tellers, deceivers' speech is either devoid of emotion language or includes more negatively valenced terms (42). But in other quarters, liars have adopted a more persuasive, pleasant stance (46). Fraudulent responses during a quarterly earnings call included more extremely pleasant adverbs and adjectives (36). Again, context is a critical guide as to whether liars might be motivated to paint a rosy picture.

## Implications for Deception Detection in the Courtroom Context

The complexities of deception indicators might lead one just to rely on gut judgments of veracity. That has its merits (47). But there are still ways to separate the truthful wheat from the deceptive chaff. Signs of a frozen demeanor, occasionally peppered with face-and lip-adaptors, invite a closer look, particularly earlier during testimony. Close attention to voice and language choices that are not easily feigned can be particularly revealing. Comparing what are likely prepared or rehearsed remarks to extemporaneous ones will expose the most revelatory verbal and non-verbal indicators. And, questioning strategies that require multiple retellings of a narrative can further draw out behavior to be analyzed.

In sum, discoveries from emerging detection technologies and interviewing methods represent a new torch illuminating the search for the truth, the whole truth and nothing but the truth.

## Author Contributions

The author confirms being the sole contributor of this work and has approved it for publication.

### Conflict of Interest Statement

The author declares she is affiliated with Discern Science International, a for-profit entity that develops systems for credibility assessment.
